# Comparative genome-wide association studies of a depressive symptom phenotype in a repeated measures setting by race/ethnicity in the multi-ethnic study of atherosclerosis

**DOI:** 10.1186/s12863-015-0274-0

**Published:** 2015-10-12

**Authors:** Erin B. Ware, Bhramar Mukherjee, Yan V. Sun, Ana V. Diez-Roux, Sharon L.R. Kardia, Jennifer A. Smith

**Affiliations:** Department of Epidemiology, University of Michigan, Ann Arbor, MI USA; Institute of Social Research, University of Michigan, 1415 Washington Heights #4614, Ann Arbor, MI 48109 USA; Department of Biostatistics, University of Michigan, Ann Arbor, MI USA; Department of Epidemiology, Emory University, Atlanta, GA USA; Department of Epidemiology and Biostatistics, Drexel University, Philadelphia, PA USA

**Keywords:** Depressive symptoms, Generalized estimating equations, Genome-wide association studies, Longitudinal, Psychogenetics

## Abstract

**Background:**

Time-varying phenotypes have been studied less frequently in the context of genome-wide analyses across ethnicities, particularly for mood disorders. This study uses genome-wide association studies of depressive symptoms in a longitudinal framework and across multiple ethnicities to find common variants for depressive symptoms. Ethnicity-specific GWAS for depressive symptoms were conducted using three approaches: a baseline measure, longitudinal measures averaged over time, and a repeated measures analysis. We then used meta-analysis to jointly analyze the results across ethnicities within the Multi-ethnic Study of Atherosclerosis (MESA, n = 6,335), and then within ethnicity, across MESA and a sample from the Health and Retirement Study African- and European-Americans (HRS, n = 10,163).

**Methods:**

This study uses genome-wide association studies of depressive symptoms in a longitudinal framework and across multiple ethnicities to find common variants for depressive symptoms. Ethnicity-specific GWAS for depressive symptoms were conducted using three approaches: a baseline measure, longitudinal measures averaged over time, and a repeated measures analysis. We then used meta-analysis to jointly analyze the results across ethnicities within the Multi-ethnic Study of Atherosclerosis (MESA, n = 6,335), and then within ethnicity, across MESA and a sample from the Health and Retirement Study African- and European-Americans (HRS, n = 10,163).

**Results:**

Several novel variants were identified at the genome-wide suggestive level (5×10^−8^ < *p*-value ≤ 5×10^−6^) in each ethnicity for each approach to analyzing depressive symptoms. The repeated measures analyses resulted in typically smaller p-values and an increase in the number of single-nucleotide polymorphisms (SNP) reaching genome-wide suggestive level.

**Conclusions:**

For phenotypes that vary over time, the detection of genetic predictors may be enhanced by repeated measures analyses.

**Electronic supplementary material:**

The online version of this article (doi:10.1186/s12863-015-0274-0) contains supplementary material, which is available to authorized users.

## Background

With advances in the ability of statistical software to handle data with repeated measures, longitudinal data analysis is becoming more feasible in genetic association studies. While these analyses are more complicated and computationally intensive than analyses using only baseline measures, longitudinal data has been used to identify variants that influence complex traits above and beyond that of cross-sectional measurements [1]. Because depressive symptoms may vary over time in relation to a variety of circumstantial factors, repeated measures of depressive symptoms may provide a better characterization of an individual’s phenotype than a single measure, thus increasing power to detect genetic susceptibility loci.

There are a number of circumstances where longitudinal data analysis may be more informative or powerful than cross-sectional analyses based on single or time averaged measures. If there is substantial variability over time in the outcome or interaction of other covariates or SNPs with time, a longitudinal analysis will clearly be more informative [2]. For a given fixed number of observations, cross sectional analyses will be more powerful than repeated measures in the presence of within-subject correlations (e.g. cross sectional n = 500; repeated measures n = 250 with two measures), but longitudinal analyses permits detection of factors associated with within person changes over time, which often allows stronger causal inferences [2]. A genetic association analysis with longitudinal data also follows these well-established properties, except for the fact that the analysis is repeated millions of times and tail behavior of the test statistics along with robustness issues become more critical since much smaller significance thresholds are used than traditional inference at a 5 % level of significance.

Depressive symptoms exist on a spectrum, varying in both severity and duration, and are often measured in population-based studies using the 20-item Center for Epidemiological Studies Depression scale (CES-D). Given the benefits of longitudinal analysis, the ability to detect genetic predictors of depression may be enhanced by analyzing depressive symptoms both over time and quantitatively [3], rather than applying cutoffs or defining disorders like Major Depressive Disorder (MDD) at the extreme of the continuum for a single time point [4].

The Multi-Ethnic Study of Atherosclerosis (MESA) European sub-sample was recently part of a discovery sample for a cross-sectional genome-wide association study (GWAS) of depressive symptoms conducted by the Cohorts for Heart and Aging Research in Genomic Epidemiology (CHARGE) consortium [5]. This GWAS focused on a single measure of depressive symptoms (as assessed by CES-D) in individuals of European descent. Though no loci reached genome-wide significance in the discovery sample (composed of 34,549 individuals), one of the seven most significant SNPs had a suggestive association in the replication sample (rs161645, 5q21, *p* = 9.19×10^−3^). This SNP reached genome-wide significance (*p* = 4.78×10^−8^) in overall meta-analysis of the combined discovery and replication samples (n = 51,258) [5]. Important limitations of this GWAS include the reliance on a single measure of depressive symptoms and the focus on a single race/ethnic group.

In the present study, we use longitudinal data on a continuous measure of depressive symptoms collected over a 9 year period from three exams in MESA to conduct GWAS on depressive symptoms in four race/ethnicities. We also contrast different approaches of incorporating the repeated measures into the GWAS: (1) analyzing a single time-point measure (baseline), (2) averaging measures over time, and (3) conducting a repeated measures outcome analyses. Finally, we jointly analyze repeated measures GWAS results from MESA and up to ten exams from the Health and Retirement Study. The MESA study includes a total of 650, 507, and 5,178 participants with one, two, and three measures, respectively, while the HRS sample consists of 34, 147, and 9,982 individuals with one, two, and three-plus measures, respectively) in an overall meta-analysis for European Americans and African Americans to increase power. To our knowledge, there have been no GWAS of repeated measures of depressive symptoms measured over time in individuals of multiple race/ethnicities.

## Results

### Descriptive statistics

Descriptive statistics for MESA and HRS are presented in Table 1. The MESA sample includes 6,335 individuals (48 % male). Mean age at baseline is 62.2 years and approximately 40 %, 25 %, 12 %, and 23 % are of European (EA), African (AA), Chinese (CA), and Hispanic (HA) American self-reported ethnicity, respectively.

**Table 1:** Descriptive statistics

	MESA^1^								HRS^2^			
	n = 6,335								n = 10,163			
	European American		African American		Hispanic American		Chinese American		European American		African American	
	n = 2,514		n = 1,603		n = 1,443		n = 775		n = 8,652		n = 1,511	
	Mean	(SD)	Mean	(SD)	Mean	(SD)	Mean	(SD)	Mean	(SD)	Mean	(SD)
Depression score^3^												
Baseline CES-D^4^	8	(7.8)	7.6	(7.6)	9.9	(9.2)	6.3	(6.6)	1.2	(1.8)	1.2	(1.8)
Averaged CES-D	8.7	(7.4)	7.8	(6.7)	10.2	(8.5)	6.2	(5.6)	1.2	(1.3)	1.9	(1.6)
Age	62.6	(10.2)	62.2	(10.1)	61.4	(10.3)	62.4	(10.4)	58.4	(8.8)	56.8	(8.2)
Sex (%)	n	%	n	%	n	%	n	%	n	%	n	%
Male	1207	48.0	744	46.4	711	49.3	385	49.7	3,556	41.1	639	42.3
Site (%)												
Baltimore, MD	505	20.1	482	30.1	-	-	-	-	-	-	-	-
Chicago, IL	526	20.9	258	16.1	-	-	275	35.4	-	-	-	-
Forsyth County, NC	548	21.8	425	26.5	3	0.2	-	-	-	-	-	-
Los Angeles, CA	133	5.3	143	8.9	554	38.4	498	64.3	-	-	-	-
New York, NY	209	8.3	295	18.4	431	29.9	2	0.3	-	-	-	-
St. Paul, MN	593	23.6	-	-	455	31.5	-	-	-	-	-	-
Anti-depressant Use (%)	307	12.2	61	3.8	84	5.8	19	2.5	-	-	-	-
Intraclass correlation												
Repeated Measures CES-D	0.60		0.44		0.57		0.57		0.48		0.51	

^1^Multi-Ethnic Study of Atherosclerosis, ^2^Health and Retirement Study, ^3^CES-D measured as 20-item sum in MESA and as 8-item sum in HRS, ^4^Center for Epidemiologic Studies - Depression

In MESA, the mean baseline depressive symptom score ranged from 6.3 (standard deviation (SD): 6.6) in the CA subsample to 9.9 (SD: 9.2) in the HA subsample out of a possible score of 60. CES-D scores increased over time in the EA (linear trend model for exam: β_exam_ = 0.25, *p* < 0.0001), AA (β_exam_ = 0.03, *p* = 0.67), and HA (β_exam_ = 0.13, *p* = 0.11) sub-groups, but this increase in trend was only significant in EA. The CA sub-group showed a non-significant decrease in depressive symptom score over time (β_exam_ = −0.04, *p* = 0.67). The intraclass correlation (within-person correlation) across all exams for which an individual had a valid CES-D score (up to three time-points) ranged from 0.44 in AA to 0.60 in EA.

The HRS analysis sample contains 10,163 respondents (41 % male), with 8,652 EA (85 %) and 1,511 AA (15 %). Mean age at baseline was 58 years. The CES-D8 depressive symptom score in HRS EA increased significantly over study waves (β_exam_ = 0.03, *p* < 0.0001) and decreased significantly in AA participants over time (β_exam_ = −0.01, *p* = 0.04). The intraclass correlation for the HRS participants across exams was 0.48 for EA participants and 0.51 for AA participants.

### Ethnicity-specific association analysis in MESA

Table 2 shows the number of SNPs, minimum *p*-value of the adjusted association between SNP dosage and outcome, and the genomic-control inflation factor, lambda, for each ethnicity in MESA and HRS. QQ plots are available in Additional file 1. The inflation factor, the extent to which the chi-square statistic is inflated due to confounding by ethnicity [6], is very close to 1.0 for all analyses, indicating adequate adjustment for population structure. One SNP reached the genome-wide significant threshold in the HA subset in the baseline CES-D approach in the intronic region of the *MUC13* gene (rs1127233, 3q22.1, β = 0.2382, *p*-value = 3.85×10^−8^; averaged β = 0.1598, *p*-value = 9.23×10^−6^; repeat measures β = 0.1753, *p*-value = 2.06×10^−6^). This gene has previously been associated with cancer pathogenesis (e.g. [7–16]) but has not been implicated in any psychiatric disorders. This SNP was not associated with CES-D in the other race/ethnicities nor did it show consistent direction across ethnicity in the baseline CES-D analyses (AA: β = −0.0112, *p*-value = 0.7707; EA: β = −0.0228, *p*-value = 0.4527; CA: β = 0.0562, *p*-value = 0.4351). There were no other genome-wide significant SNPs in any of the ethnicities for any of the baseline, average, and repeated-measures modeling approaches though there were many suggestive *p* < 10^−6^ findings.

**Table 2:** Minimum p-value from GWAS of baseline, averaged, and repeated measures of CES-D^1^ across ethnicities, MESA^2^ and HRS^3^

			Baseline CES-D score			Averaged CES-D score			Repeated measures CES-D score		
Study	Ethnicity	# of SNPS	Min	# of	λ^5^	Min	# of unique SNPs	λ	Min	# of unique SNPs	λ
			p-value	unique SNPs^4^		p-value			p-value		
MESA											
African American		2380122	2.05×10^−7^	7	1.01	6.64×10^−7^	9	1.00	1.63×10^−7^	11	1.01
European American		2166730	1.33×10^−7^	9	1.01	8.26×10^−7^	6	1.00	6.04×10^−7^	11	1.01
Chinese American		1801470	2.48×10^−6^	1	0.99	1.42×10^−6^	2	1.00	2.71×10^−7^	4	1.02
Hispanic American		2148331	3.85×10^−8^	10	1.00	1.61×10^−6^	4	1.00	9.25×10^−7^	11	1.01
HRS											
African American		2446939	-	-	-	-	-	-	2.07×10^−6^	-	1.01
European American		2177692	-	-	-	-	-	-	6.54×10^−7^	-	1.04

^1^Center for Epidemiological Studies – Depression, ^2^Multi-Ethnic Study of Atherosclerosis, ^3^Health and Retirement Study, ^4^Number of unique (independent) SNPs, linkage disequilibrium R^2^ < 0.80, INFO > 0.80, with ethnicity-specific minor allele frequency > 5 % and *p*-values < 1×10^−5^, ^5^genomic control lambda

### Comparison of results across approaches

To compare association results between the different versions of the CES-D scores, we assessed scatter plots for the p-values (*p* < 5×10^−4^) from each pair of SNPs for the baseline CES-D score compared to the averaged CES-D score phenotype (Additional file 2), the baseline CES-D score compared to the repeated measures CES-D score (Additional file 3), and the averaged CES-D score against the repeated measures CES-D score (Additional file 4) within each of the four ethnicities in MESA. For all four ethnicities, the Spearman’s rank correlations between the baseline versus averaged CES-D phenotype and between the baseline and repeated measures CES-D phenotypes ranged between 0.46 and 0.57. The correlations between p-values for the averaged versus repeated measures CES-D phenotype ranged between 0.85 and 0.92 (Table 3). We observed an increase in the number of unique (LD R^2^ < 0.8) genome-wide suggestive SNPs from baseline to repeated measures for each ethnicity (EA: eight to nine; AA: four to 11; CA: one to four; HA: six to ten), with some (at least two SNPs appearing in multiple approaches as genome-wide suggestive within each ethnicity) consistency in the SNPs across approach (Additional file 5).

**Table 3:** Spearman’s correlation coefficients and 95 % confidence intervals for paired p-values in Multi-Ethnic Study of Atherosclerosis

		Baseline vs averaged CES-D score	Baseline vs repeated measures CES-D score	Averaged vs repeated measures CES-D score
		r, (95 % Confidence interval)	r, (95 % Confidence interval)	r, (95 % Confidence interval)
MESA	African American	0.53, (0.53, 0.53)	0.54, (0.54, 0.54)	0.88, (0.88, 0.88)
	European American	0.54, (0.54, 0.54)	0.57, (0.57, 0.57)	0.92, (0.92, 0.92)
	Chinese American	0.48, (0.48, 0.48)	0.46, (0.46, 0.47)	0.85, (0.85, 0.85)
	Hispanic American	0.54, (0.54, 0.54)	0.56, (0.55, 0.56)	0.88, (0.88, 0.88)

### Meta-analysis across ethnicities in MESA

The results from the three meta-analyses performed within MESA across ethnicities for the baseline, averaged, and repeated measures CES-D scores are presented in Table 4. In the table, we present every unique (LD R^2^ < 80 %) SNP with *p* < 1×10^−6^. The meta-analysis only included SNPs with ethnicity-specific minor allele frequency (MAF) > 5 % calculated within ethnicity using only MESA participants. These meta-analyses showed no genome-wide significant results. Thirteen SNPs reached a genome-wide suggestive threshold in these meta-analyses. The smallest p-value was in the repeated measures meta-analysis on chromosome 2, (rs41379347, 2q32.2, *p*-value = 1.81×10^−7^). This SNP was only present (with MAF > 5 %) in the CA and HA subsamples. This SNP is in the intronic region of the *STAT1* gene, IFN-γ transcription factor signal transducer and activator of transcription 1, previously implicated as a tumor suppressor [17, 18]. This SNP has not been previously associated with depressive symptoms.

**Table 4:** Meta-analysis results^1^ across ethnicities in MESA^2^ (*p*-values < 1×10^−5^) for each depressive symptom score modeling approach

Approach	CHR	SNP	Location	Coded allele	Coded allele frequency	Z-score	*P*-value	Direction^3^	Closest gene^4^ within ±50kB
Baseline									
	8	rs2440212	97270629	A	0.66	4.47	7.73×10^−6^	++++	(GDF6)
	9	rs13440434	131953827	A	0.87	−4.50	6.79×10^−6^	----	(GPR107)
	10	rs7087469	54339854	A	0.13	4.76	1.93×10^−6^	++?+	-
	13	rs9560521	89457392	A	0.13	4.69	2.69×10^−6^	++++	(LINC00559)
	16	rs8046816	71863525	A	0.47	4.53	5.92×10^−6^	++++	-
	20	rs17215529	3923402	A	0.85	4.79	1.66×10^−6^	++?+	RNF24
Averaged									
	1	rs3100865	2795967	T	0.49	4.44	9.02×10^−6^	++++	-
	2	rs41379347	191577187	T	0.89	−4.58	4.57×10^−6^	??--	STAT1
	2	rs7602149	114357038	T	0.84	−4.57	4.78×10^−6^	--?-	LOC728055
	2	rs13001068	182706602	A	0.92	4.50	6.95×10^−6^	? + ?+	(PDE1A)
	7	rs697521	16730681	T	0.13	−4.74	2.12×10^−6^	--?-	BZW2
	8	rs7350109	60753909	A	0.81	−4.5	6.88×10^−6^	--?-	-
	11	rs1448128	121291660	C	0.24	−4.58	4.61×10^−6^	----	-
	22	rs5760767	23696411	T	0.51	4.58	4.62×10^−6^	++++	(TMEM211)
Repeated measures									
	1	rs11590206	145665933	A	0.16	−4.72	2.33×10^−6^	----	(GJA5)
	2	rs41379347	191577187	T	0.89	−5.22	1.81×10^−7^	??--	STAT1
	2	rs7602149	114357038	T	0.84	−4.62	3.83×10^−6^	--?-	LOC728055
	4	rs13139186	96637940	T	0.90	−4.48	7.44×10^−6^	----	UNC5C
	4	rs233976	104823918	A	0.21	4.47	7.75×10^−6^	?+++	TACR3
	7	rs11771332	86539742	A	0.81	−4.48	7.45×10^−6^	?-?-	(KIAA1324L)
	9	rs2211185	1332721	T	0.77	4.55	5.42×10^−6^	++++	-
	18	rs2728505	21474070	A	0.55	−4.47	7.84×10^−6^	----	-
	22	rs5760767	23696411	T	0.51	4.54	5.68×10^−6^	++++	(TMEM211)

^1^filtered at ethnicity-specific minor allele frequency 5 %, where the SNP was present in at least two ethnicities, linkage disequilibrium R^2^ < 80 %, and heterogeneity *p*-value ≥ 0.1; ^2^Multi-Ethnic Study of Atherosclerosis; ^3^Order corresponding to direction positions: African, European, Chinese, Hispanic American; ^4^parentheses indicate location outside of gene

### Joint-analysis across studies for EA and AA

Results from the joint-analyses (MESA + HRS) for EA and AA, separately, are presented in Table 5. While no SNP reached the genome-wide level, eight SNPs (EA n = 3; AA n = 5) satisfied the suggestive threshold for significance. In EA the smallest p-value (rs6842756, 4q35.1, *p*-value = 6.54×10^−7^) was located within the *ENPP6* gene, which is expressed primarily in the kidney and brain and has not been implicated in any disorders or diseases [http://omim.org/]. In AA the smallest observed *p*-value (rs2426733, 20q13.31, *p*-value = 2.07×10^−6^) was located downstream of the *RBM38* oncogene. *RBM38* encodes an RNA binding protein found to regulate *MDM2* (12q14.3-q15) gene expression through mRNA stability [19, 20], but has not been identified in genetic studies of psychiatric disorders [17] (http://omim.org/).

**Table 5:** Meta-analysis results^1^ between MESA^2^ and HRS^3^ (*p*-values < 1×10^−5^) for repeated measures depressive symptom score GEE analyses

Race	CHR	SNP	Location	Coded allele	Coded allele frequency	Z-score	P-value	Direction^4,5^	Closest gene^6^ within ±50kB
African American									
	1	rs10776776	114384683	T	0.55	4.73	2.30×10^−6^	++	(SYT6)
	1	rs1417303	235193008	T	0.59	−4.43	9.46×10^−6^	--	LOC440737
	2	rs4629180	101454802	A	0.83	−4.51	6.41×10^−6^	--	(LOC731220)
	2	rs6711630	126534599	T	0.93	4.58	4.70×10^−6^	++	
	7	rs10249133	12514004	T	0.39	−4.47	7.67×10^−6^	--	(LOC100133035)
	8	rs17067630	3661853	A	0.85	4.70	2.57×10^−6^	++	CSMD1
	11	rs11036016	40661316	A	0.80	4.68	2.94×10^−6^	++	LRRC4C
	15	rs4551976	49264445	T	0.63	−4.45	8.48×10^−6^	--	(CYP19A1)
	16	rs365962	85267450	C	0.69	−4.53	5.83×10^−6^	--	(LOC101928614)
	20	rs2426733	55454729	A	0.40	−4.75	2.07×10^−6^	--	(RBM38)
European American									
	1	rs12031875	71357685	A	0.82	4.81	1.54×10^−6^	++	ZRANB2-AS2
	4	rs6842756	185341452	A	0.92	4.98	6.54×10^−7^	++	ENPP6
	6	rs6941340	16145531	T	0.48	−4.47	7.95×10^−6^	--	
	9	rs11794102	111772109	A	0.91	4.54	5.70×10^−6^	++	PALM2-AKAP2
	13	rs6492314	110267411	C	0.28	−4.75	2.00×10^−6^	--	
	16	rs12921740	20219533	T	0.51	−4.55	5.44×10^−6^	--	(GP2)
	18	rs2612547	41290709	A	0.83	4.47	7.94×10^−6^	++	SLC14A2
All samples									
	1	rs2300177	71270950	T	0.19	−4.56	5.09×10^−6^	? − +−−?	PTGER3
	1	rs1539418	145676734	A	0.16	−4.53	5.87×10^−6^	----??	
	1	rs7415169	168997688	A	0.85	4.70	2.58×10^−6^	++++++	
	2	rs6711630	126534599	T	0.93	4.58	4.70×10^−6^	+????+	
	2	rs41379347	191577187	T	0.89	−5.22	1.81×10^−7^	??--??	STAT1
	4	rs6552764	185341497	A	0.86	4.64	3.41×10^−6^	− + ? − ++	ENPP6
	8	rs12541177	124869454	T	0.31	−4.48	7.43×10^−6^	------	FAM91A1
	9	rs9408311	38704682	T	0.84	4.92	8.87×10^−7^	++ − +++	
	15	rs16975781	94245464	T	0.90	4.62	3.83×10^−6^	+++++−	
	16	rs12921740	20219533	T	0.59	−4.70	2.61×10^−6^	-−− + −−	(GP2)

^1^Filtered at ethnicity-specific minor allele frequency of 5 %, where the SNP was present in at least two ethnicities, linkage disequilibrium R^2^ < 80 %, and heterogeneity *p*-value ≥ 0.1; ^2^Multi-Ethnic Study of Atherosclerosis; ^3^Health and Retirement Study ^4^Order corresponding to direction positions: African, European, Chinese, Hispanic American; ^5^For all samples analyses, order corresponding to direction position: MESA African American, MESA European American, MESA Chinese American, MESA Hispanic American, HRS European American, HRS African American; ^6^parentheses indicate location outside of gene

### Meta-analysis across all ethnicities in MESA and HRS

For the meta-analysis across all ethnicities in both HRS and MESA, we found no SNPs reaching genome-wide significance, though we found seven SNPs reaching genome-wide suggestive thresholds (Table 5). The most strongly associated SNPs in the meta-analysis, rs41379347 (*p*-value = 1.81×10^−7^) is located on chromosome 2 (in the *STAT1* gene). The SNP rs41379347 was found previously in the MESA meta-analysis across ethnicity. This SNP was only present (with MAF > 5 %) in the MESA CA and HA samples, and thus, no new information was gained in the joint analysis across MESA and HRS.

### Consistency with previous GWAS on depressive symptom scores

There has been one published GWAS conducted on depressive symptom scores [5], for which MESA EA were part of the discovery sample. This GWAS found one genome-wide significant SNP in overall meta-analysis of 51,258 European-ancestry individuals (rs161645, 5q21, *p* = 4.78×10^−8^). In our EA subsample, p-values for this SNP in our baseline and repeated measures analysis were 0.116 and 0.055, respectively, with consistent effect directions (+) as the Hek, *et al.* [5] finding. Additionally, this SNP had a cross-ethnicity, within MESA meta-analysis p-value of 0.067 in the baseline analysis, 0.006 in the averaged CES-D analysis, and 0.008 in the repeated measures analysis. The overall direction of effect was consistent with the published GWAS for EA, AA, and HA, though the direction of effect was opposite for CA. This SNP had p-values of 0.951 and 0.113 for the cross-study (i.e. combining MESA and HRS) EA and AA analyses, respectively.

## Discussion

This is the first set of GWASs to the authors’ knowledge, to investigate common genetic variants for depressive symptoms in a longitudinal setting across four different ethnicities. We performed GWASs within each ethnicity for three different longitudinal approaches to a depressive symptom phenotype (baseline, averaged, and repeated measures) and meta-analyzed them across ethnicity and across study. Though our joint meta-analysis of all ethnicities in both studies comprises 16,498 individuals, and the power to detect genetic variants of depression has been shown to increase when assessing depression quantitatively — as opposed to using a dichotomous definition or cutoff point [21] — we did not find any variants that reached genome-wide significant levels in the European-, African-, Hispanic-, or Chinese-American, race/ethnicity-specific GWAS, in meta-analyses across ethnicity in MESA, or in joint analyses across study for the European and African Americans with any evidence of replication. However, we did find several novel variants at a genome-wide suggestive level and we observed an increase in the number of unique (LD R^2^ < 0.8) genome-wide suggestive SNPs from baseline to repeated measures for each ethnicity (Additional file 5). We have taken the single SNP that has been credibly associated with depressive symptoms from Hek *et al.*, [5] and presented evidence that a longitudinal framework may improve upon findings for depressive symptoms.

Hek, *et al.* [5] identified a SNP (rs161645) associated with a large sample of European-ancestry participants measured at a single time point. It is important to note that European Americans from MESA were used in the discovery sample for the previously published GWAS. We found that in the EA subsample, repeated measures better characterized depressive symptoms and the longitudinal analysis resulted in a repeated measures p-value for rs161645 (*p* = 0.055) less than half that of the baseline measures model (*p* = 0.116). If we consider this SNP a true signal (or proxy for a true signal), we indeed demonstrate that the p-value has decreased from the baseline to the repeated measures analysis.

A repeated measures analysis makes use of the full information content in the outcome and exposure/covariates for longitudinal data. For example, in an analysis with repeated measures data, if there is drop-out in the study and we use subject level averages, the homoscedasticity assumption of linear models is violated as different averages will be based on different number of observations and the ones with more observation will have higher precision. Averaging the exposure data may also lead to substantial loss in power. If there is a time trend or interaction of covariates (or SNPs) with time, a longitudinal model is expected to have larger power than a cross-sectional or averaged model. Longitudinal modeling is a better general framework as it allows incorporation of time-varying covariates (instead of averaging them) and allows exploration of G × E interaction in follow-up analysis with cumulative exposure trajectory. Although we saw an increase in the number of unique genome-wide suggestive SNPs for repeated measures compared to baseline, we note that since most of the SNPs are non-significant, this may be simply a comparison of false positives. However, in view of the existing literature one can argue that a longitudinal analysis is generally more efficient than using a summary quantity in the presence of repeated measures data.

For repeated measures, there are multiple modeling approaches. GEE produces unbiased and consistent estimates of the fixed effect parameters, even under misspecification of the correlation structure. Also, if the correlation structure is correctly specified, there is gain in terms of efficiency. GEE can be argued as a better framework than a linear regression model in terms of its robust estimates of the standard error and behavior of QQ plots as it protects under model misspecification [22]. That is why we chose the GEE framework for this large-scale association analysis instead of an alternative linear mixed model analysis.

Though GWAS have been used for over a decade, most variants identified for diseases have had very modest effect sizes, often explaining less than 1 % of the variance of quantitative traits [23]. Because of the small effect sizes, very large sample sizes are required to reach adequate power to detect genetic effects and produce reliable inferences [24]. Preliminary steps have been taken to increase power in our study through the characterization of a longitudinal phenotype. Most individual studies, including this one, are underpowered to detect these variants and often collaboration across many studies, involving meta-analysis, are used to increase sample size, and thus power [23, 25]. Though this framework is frequently used for common traits with standard measures, it is exceedingly difficult to find studies measuring depressive symptoms using the CES-D in multiple ethnicities, across time.

The depressive symptom GWAS literature to date includes one GWAS, with only one genome-wide significant result [5]. The literature for similar phenotypes, such as Major Depressive Disorder (MDD), has nine GWAS studies [26–34], a mega-analysis of the nine GWAS that included almost 19,000 European unrelated individuals [35], and a recent low-coverage, whole-genome sequencing analysis in the Chinese ethnicity [36]. Only two loci reached genome wide significance in individual studies [28, 37], but these loci were not significantly associated with MDD in the meta-analysis [35]. The whole-genome sequencing analysis, using a joint discovery-replication analysis and linear mixed models including a genetic relatedness matrix as a random effect, identified two loci on chromosome 10, one near the *SIRT1* gene (*p* = 2.53×10^−10^) and the other in an intron of the *LHPP* gene (*p* = 6.45×10^−12^) [36]. Meta-analyses of genetic predictors of MDD (up to early 2015) are currently consistent with chance findings and hypothesized candidate genes identified from physiological pathways (such as *TPH2*, *HTR2A*, *MAOA*, *COMT*) have rarely been identified/replicated as predictors of MDD in GWAS [34, 38–40]. Accordingly, we did not find a significant association with depressive symptoms for the SNPs that reached genome-wide significance in MDD GWAS nor those in hypothesized candidate genes. However, whole-genome sequencing and statistical modeling alternatives to traditional linear regression provide a promising avenue for discovering new genes that influence depressive illness, and follow-up of these new regions will be imperative.

One potentially important reason that SNPs detected through GWAS and biological candidate genes rarely replicate is because despite the CES-D correlating strongly with depression and having been used in hundreds of studies, the CES-D is not a diagnostic tool. The CES-D only measures depressive symptoms over the past week. The MESA study exams were spaced approximately 12 – 24 months apart (the HRS surveys 24 months apart). It is possible that failure to capture changes in depressive symptoms between the assessments introduced measurement error in the phenotype. Additionally, in the baseline and repeated measures analyses, though log-transformed to improve normality, the distribution of CES-D still deviated from the normal distribution. This is a consistent limitation of CES-D scores in the literature, and it should be noted that the p-values from our baseline and repeated measures models may reflect the non-normal distribution of the phenotype.

We included only common variants (those with ethnicity-specific MAF > 5 %) in our analysis. One reason we may not have found any significant genetic variants of depressive symptoms is that we did not look at rare variants or copy number variants. New methods for analyzing rare variants or SNP sets, such as Sequence Kernel Association Testing (SKAT), are being developed and applied and may help to further elucidate genetic predictors of depressive symptoms at a gene-level and across ethnicities [41]. Additionally, it is possible that multiple SNPs with small effects, working in concert, could affect individual susceptibility to depression and depressive symptoms [42]. Further, no interactions (gene-gene or gene-environment) were evaluated in these analyses, which may play an important role in revealing the pathogenesis of depression and depressive symptoms.

## Conclusion

Since combining genetic information across ethnicities can result in false-positive findings from population stratification within genetically distinct populations, we conducted GWASs separately by ethnicity adjusting for ethnicity-specific principal components and filtered initial GWAS results by ethnicity-specific minor alleles to remove low frequency variants for more robust findings. The meta-analysis software accounts for both magnitude and direction of effect when combining information across studies (in this case different ethnicities) which is especially appropriate when studies contain differences in ethnicity, phenotype distribution, gender or constraints in sharing of individual level data [43].

Identifying genes that are associated with depression has tremendous potential to transform our understanding and treatment of depression. Utilizing longitudinal measures in GWA studies for depressive symptoms allows researchers to get a better picture of depression over the life-course. Though this study did not find any gene variants that reached genome-wide significance in the repeated measures approach, it provides a first step in examining depressive symptoms in different longitudinal settings and also across multiple ethnicities.

## Methods

### Discovery sample

MESA is a longitudinal study supported by NHLBI with the overall goal of identifying risk factors for subclinical atherosclerosis [44]. The MESA cohort (N = 6,814) was recruited in 2000–2002 from six Field Centers in Baltimore, MD; Chicago, IL; Forsyth County, NC; Los Angeles, CA; New York, NY; and St. Paul, MN. MESA participants were 45–84 years of age and free of clinical cardiovascular disease at baseline. Participants attended a baseline examination and three additional follow-up examinations approximately 18–24 months apart. At each clinic visit, participants completed a series of demographic, personal history, medical history, access to care, behavioral, and psychosocial questionnaires in English, Spanish, or Chinese. Depressive symptoms were assessed using the Center for Epidemiologic Studies Depression scale (CES-D) at exams 1, 3 and 4. The total number of participants and the corresponding response rates (of participants alive) were: exam 1 (n = 6,814), exam 2 (n = 6,239, 92 %), exam 3 (n = 5,946, 89 %), exam 4 (n = 5,704, 87 %). After removing participants with missing genetic data, depressive symptom score, or covariates used for analysis, the final sample size was 6,335 individuals (European (EA): 2,514; African (AA): 1,603; Chinese (CA): 775; Hispanic (HA): 1,443). Data supporting the results of this article are available in the dbGaP repository, phs000209.v12.p3, http://www.ncbi.nlm.nih.gov/projects/gap/cgi-bin/study.cgi?study_id=phs000209.v12.p3. Written informed consent was obtained from participants after the procedure had been fully explained and institutional review boards at each site approved study protocol (University of Minnesota Human Subjects Committee Institutional Review Board (IRB), Johns Hopkins Office of Human Subjects Research IRB, University of California Los Angeles Office for the Protection of Research Subjects IRB, Northwestern University Office for the Protection of Research Subjects IRB, Wake Forest University Office of Research IRB, Columbia University IRB).

### Depressive symptom score

Depressive symptom score was assessed using the 20-item CES-D Scale [45], which was for use in general population surveys [45, 46]. The CES-D has an excellent internal consistency (Cronbach’s alpha = 0.90) [45], and assesses depressive symptoms at a specific period in time (over the past week). The outcome measure for this analysis is a sum of the 20 items, ranging from 0 to 60. If more than 5 items were missing, the CES-D score was not calculated. If 1–5 items were missing, the scores were summed for completed items, dividing the sum by the number of questions answered and then multiplying by 20. There were 5,178 (81.7 %) participants with three measures of CES-D, 507 (8.0 %) with two measures, and 650 (10.3 %) with only baseline CES-D measures, for a total of 17,198 observations. We corrected for anti-depressant use through a similar algorithm to adjusting blood pressure for persons taking anti-hypertensive medication [5]. Detailed methods are described in Additional file 6. After adjustment for anti-depressant use, CES-D scores were log-transformed to improve normality.

### Genotyping

Approximately one million SNPs were genotyped using the Affymetrix Genome-Wide Human SNP Array 6.0. Imputation was performed using the IMPUTE 2.1.0 program in conjunction with HapMap Phase I and II reference panels (CEU + YRI + CHB + JPT, release 22 - NCBI Build 36 for AA, CA, and HA participants; CEU, release 24 - NCBI Build 36 for EA). Imputation SNPs were filtered at an INFO score of 0.80. We accounted for population substructure by including the top four ethnicity-specific principal components (estimated from genome-wide data) as adjustment covariates in all analyses, as proposed previously by MESA investigators and elsewhere [47, 48].

### Joint sample

The Health and Retirement Study (HRS) was used as a joint sample to be combined with MESA GWAS results in a meta-analysis [49]. These two studies have comparable participants, and similar measures of phenotype. The HRS surveys a representative sample of more than 26,000 Americans over the age of 50 every two years starting in 1992. HRS data includes information on depressive symptoms measured with a short form of the CES-D, the CES-D8. The CES-D8 includes a subset of eight items from the full 20-item CES-D [45]. The depression score for each participant was composed of the total number of affirmative depression answers. The HRS depression symptom score ranges from 0 to 8. Participants missing two or more of the eight items were excluded from the analyses. Written informed consent was obtained and the IRB at the University of Michigan approved study protocol before data collection.

Over 12,000 HRS participants were genotyped for about 2.5 million SNPs using the Illumina Human Omni-2.5 Quad beadchip. Genotypes were imputed for EA and AA using MACH software (HapMap Phase II, release #22, CEU panel for EA and CEU + YRI panel for African Americans). Imputation SNPs were filtered at an INFO score of 0.80. We accounted for population substructure by including the top four ethnicity-specific principal components (estimated from genome-wide data) as adjustment covariates in all analyses. There were 10,163 HRS participants after removing those with missing outcome, covariate or genetic information. A total of 34 (0.3 %) had only one measure of CES-D8, 147 (1.4 %) had two measures, and 9,982 (98.2 %) had three or more CES-D8 measures, for a total of 72,273 observations.

### Genome-wide association analysis

We contrasted GWAS results using different approaches to incorporate the time-varying phenotypic data: using a single (baseline) measure, taking the average across exams, or conducting a repeated measures analysis that accounts for correlation of responses within individuals.

Baseline and averaged GWA studies were analyzed using a one-step linear regression approach, adjusting for age, sex, site (in MESA) and the first four genome-wide principal components, stratified by race in PLINK v.1.07 [50, 51]. Each SNP was analyzed separately, using SNP dosages, in an additive genetic model.

For the repeated measures, we used generalized estimating equations (GEE) to account for within-individual correlations between repeated CES-D measures [52]. Within the ‘geepack’ package in the R software, we used an exchangeable (compound symmetric) correlation structure because empirical correlations for CES-D measures for exam 1, 3, and 4 were similar and we saw no significant trend in CES-D over time for any ethnicity except for the EA sub-sample [53, 54].

### Comparison of p-values across phenotype approach

To examine whether p-values from GWAS in MESA were consistent in rank across the three analysis approaches (baseline, averaged across exams, repeated measures), we calculated Spearman’s correlations between the ranks of p-values for SNP-phenotype associations within ethnic group.

### Meta-analysis

To increase statistical power to detect SNP association, we performed a fixed-effects meta-analysis combining results across all four ethnicities within the MESA study for each of the three phenotype definitions (baseline, averaged, repeated measures), weighting by sample size. In order to further investigate consistency of associations across different studies we also conducted a meta-analysis for EA and AA (separately) across the MESA and HRS studies for the repeated measures phenotype. We use only the AA and EA samples due to the availability of a large enough sample size for these two ethnicities in HRS. Finally, we performed a meta-analysis across all ethnicities and all studies to further elucidate any genetic variants across ethnicity. For the analysis that includes both MESA and HRS, the repeated measures phenotype was selected to allow for maximum power. All meta-analyses were performed using METAL [43].

### Availability of supporting data

Data supporting the results of this article are available in the dbGap repository, phs000209.v12.p3, http://www.ncbi.nlm.nih.gov/projects/gap/cgi-bin/study.cgi?study_id=phs000209.v12.p3.

## Additional files

Additional file 1:
**QQ plot of** ***p*** **-values from GWA analyses adjusted for age, sex, study site and top four principal components, ethnicity-specific minor allele frequency greater than 5 %.** (PDF 369 kb) Additional file 2:
**Comparison of p-values (** ***p*** **-value < 5×10** ^**−4**^ **) for genome-wide association studies for baseline CES-D score compared to averaged CES-D score.** CES-D: Center for Epidemiological Studies – Depression, (a) African Americans, (b) European Americans, (c) Chinese Americans, (d) Hispanic Americans. (EPS 1757 kb) Additional file 3:
**Comparison of** ***p*** **-values (** ***p*** **-value < 5×10** ^**−4**^ **) for genome-wide association studies for baseline CES-D score compared to repeated measures CES-D score.** CES-D: Center for Epidemiological Studies – Depression, (a) African Americans, (b) European Americans, (c) Chinese Americans, (d) Hispanic Americans. (EPS 1450 kb) Additional file 4:
**Comparison of** ***p*** **-values (** ***p*** **-value < 5×10** ^**−4**^ **) for genome-wide association studies for averaged CES-D score compared to repeated measures CES-D score.** CES-D: Center for Epidemiological Studies – Depression, (a) African Americans, (b) European Americans, (c) Chinese Americans, (d) Hispanic Americans. (EPS 1424 kb) Additional file 5:
**Individual SNP information for unique SNPs reaching genome-wide suggestive** ***p*** **-value threshold for MESA ethnicity-specific GWAS analyses for each methodological approach (MAF > 5 %, INFO > 0.8, LD R** ^**2**^ **< 0.80).** (PDF 110 kb) Additional file 6:
**Methodological information on anti-depressant adjustment.** (PDF 269 kb)
