# Supplementary material for: Comparative genome-wide association studies of a depressive symptom phenotype in a repeated measures setting by race/ethnicity in the multi-ethnic study of atherosclerosis
Source: BMC Genet. 2015 Oct 12;16:118. doi: 10.1186/s12863-015-0274-0 (PMC4603946; doi:10.1186/s12863-015-0274-0)

QQ plot of p-values from GWA analyses adjusted for age, sex, study site and top four principal components, ethnicity-specific minor allele frequency greater than 5%

Baseline CES-D	Averaged CES-D	Repeated measures CES-D

African American

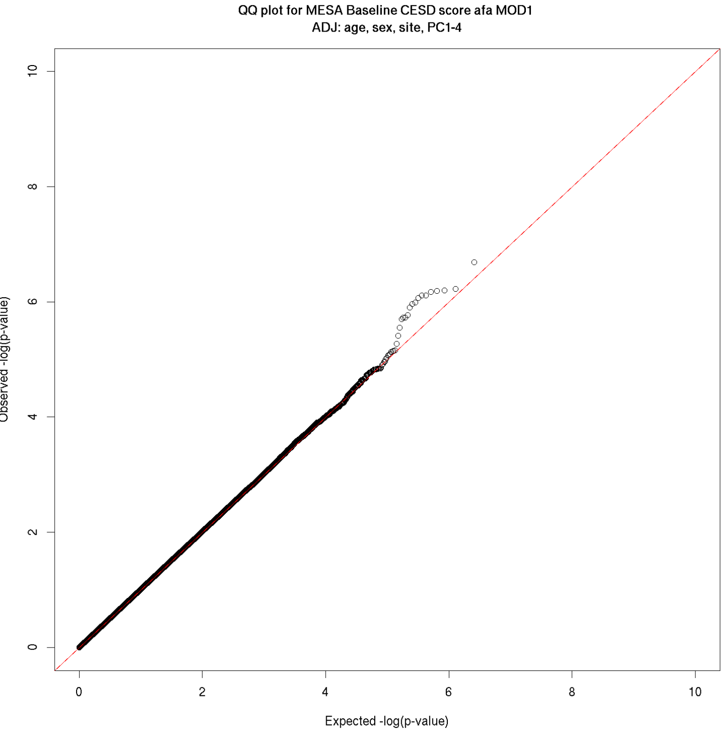

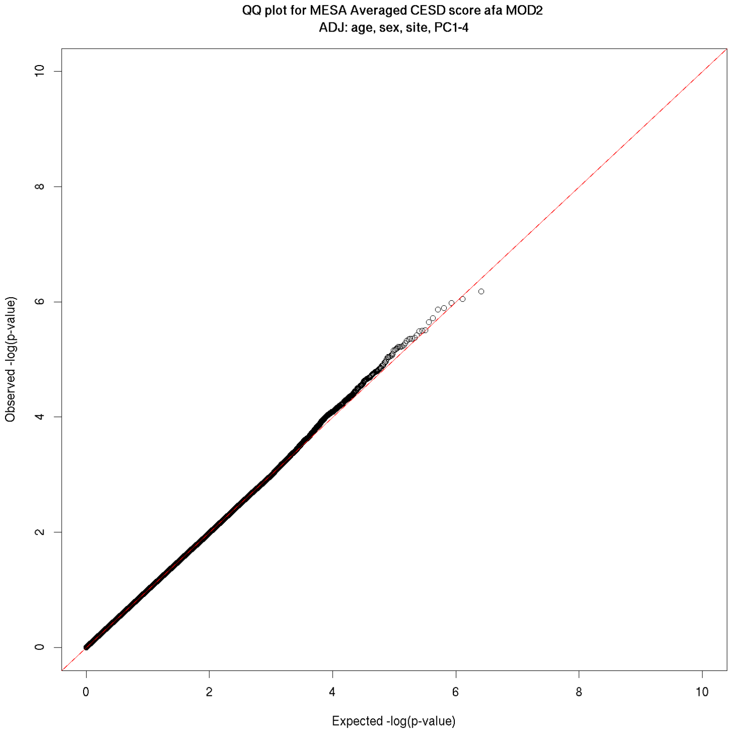

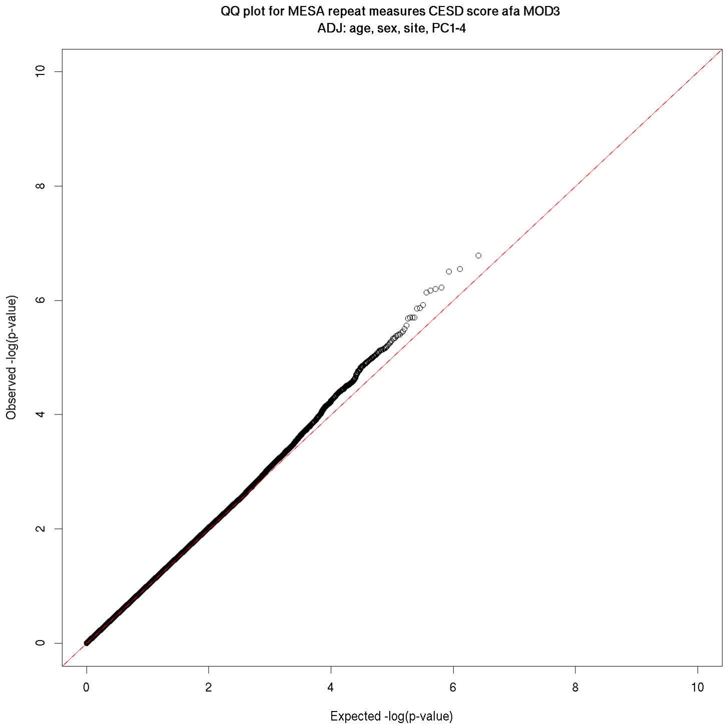

European American

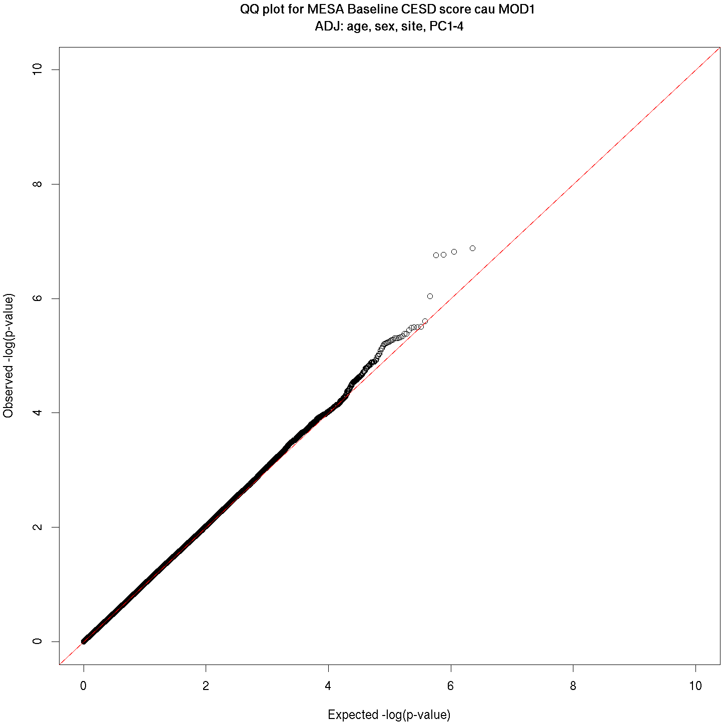

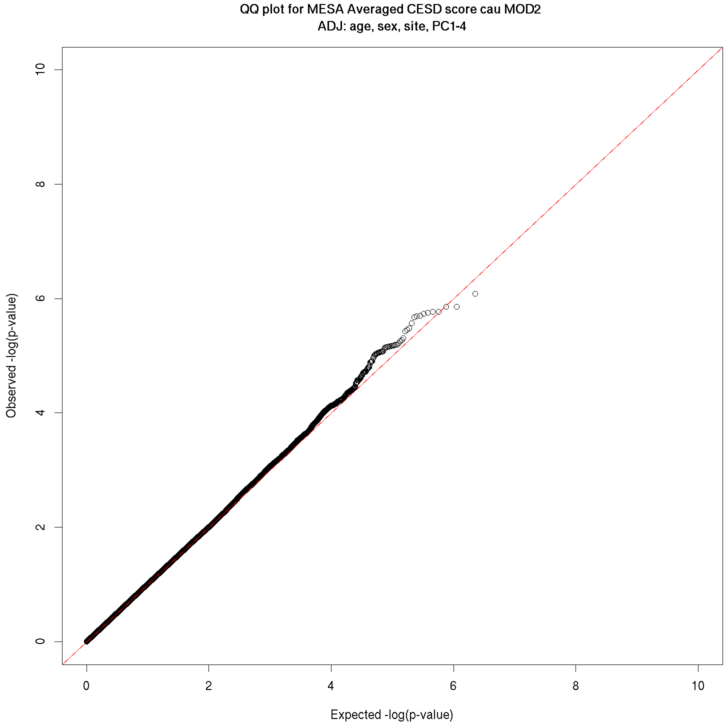

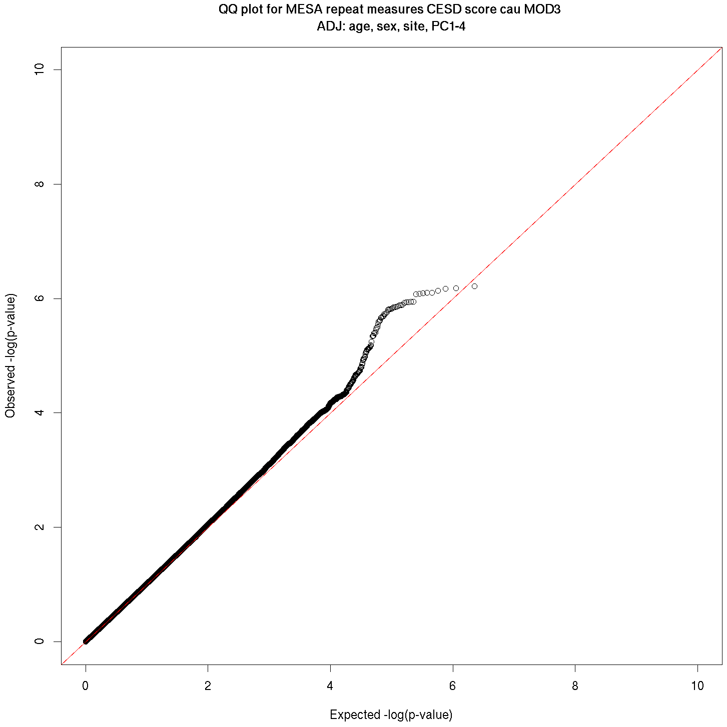

QQ plot of p-values from GWA analyses adjusted for age, sex, study site and top four principal components, ethnicity-specific minor allele frequency greater than 5%

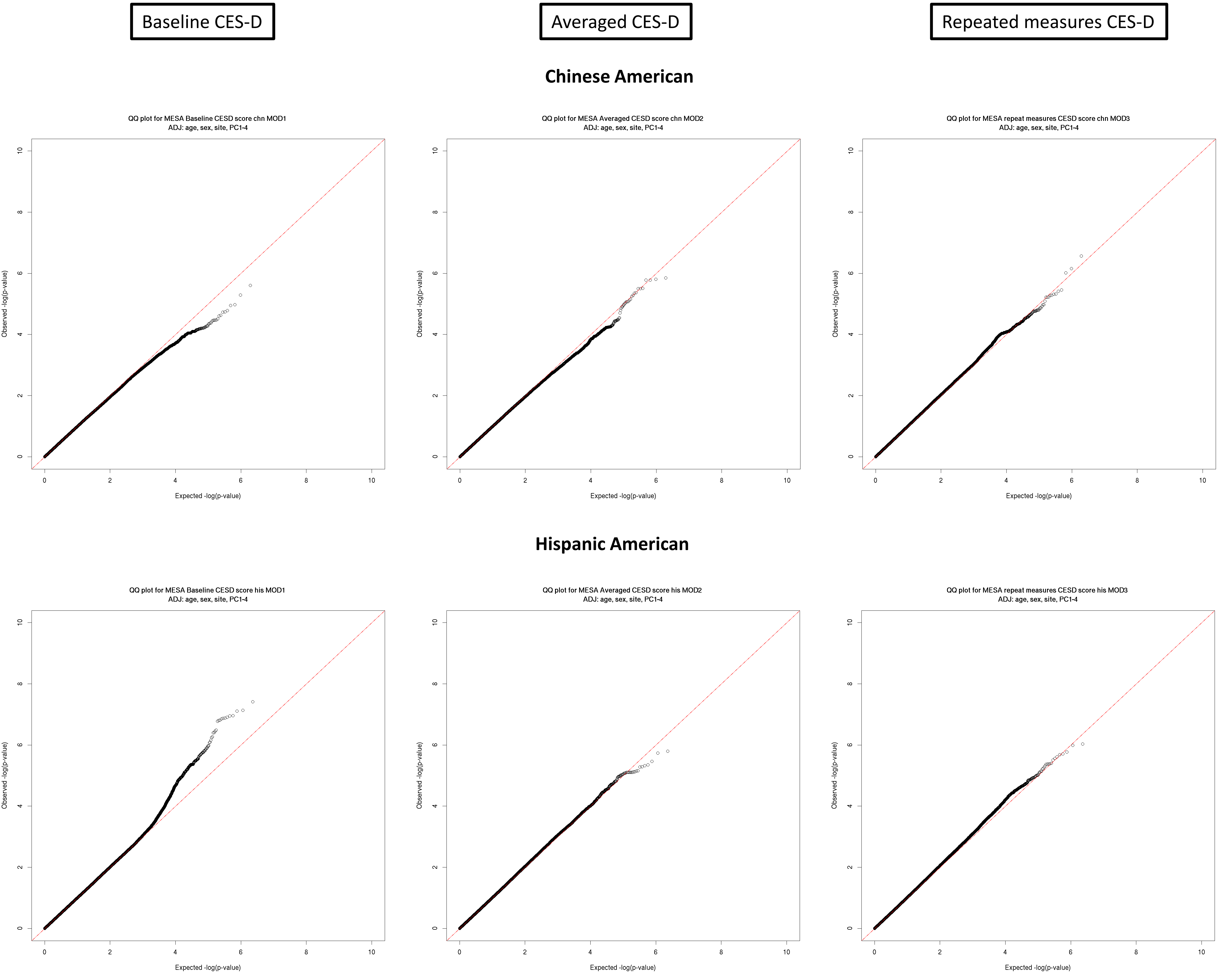

Supplement: Additional file 1: — QQ plot of p -values from GWA analyses adjusted for age, sex, study site and top four principal components, ethnicity-specific minor allele frequency greater than 5 %. (PDF 369 kb) [file 12863_2015_274_MOESM1_ESM.pdf]
