# Supplementary material for: Comparative genome-wide association studies of a depressive symptom phenotype in a repeated measures setting by race/ethnicity in the multi-ethnic study of atherosclerosis
Source: BMC Genet. 2015 Oct 12;16:118. doi: 10.1186/s12863-015-0274-0 (PMC4603946; doi:10.1186/s12863-015-0274-0)
Supplement: Additional file 5: — Individual SNP information for unique SNPs reaching genome-wide suggestive p -value threshold for MESA ethnicity-specific GWAS analyses for each methodological approach (MAF > 5 %, INFO > 0.8, LD R 2 < 0.80). (PDF 110 kb) [file 12863_2015_274_MOESM5_ESM.pdf]

Ancestry	Approach	CHR	SNP		BP	A1	A2	FRQ	INFO	Beta	SE	Pval
**African**												
	**Baseline**	2	rs17367970		14976634	A	G	0.82	0.97	0.21	0.04	1.88x10^{-6}
		3	rs2639227		81209252	C	T	0.35	0.97	-0.18	0.04	6.73x10-^{7}
		6	rs9463613		50575789	C	G	0.95	0.84	-0.40	0.08	1.70x10^{-6}
		7	rs5018071		122362272	A	G	0.71	0.99	0.18	0.04	5.97x10^{-7}
	**Averaged**	2	rs13394665	*	64244935	A	C	0.73	0.97	0.15	0.03	1.27x10^{-6}
		2	rs1069256	*	173881973	C	T	0.75	1.02	0.15	0.03	1.36x10^{-6}
		3	rs2639228		81208798	A	G	0.68	0.99	0.15	0.03	6.64x10^{-7}
		6	rs7775991		47008467	C	T	0.73	1.02	0.14	0.03	4.78x10^{-6}
		6	rs280312		50712854	G	T	0.95	1.02	-0.29	0.06	1.90x10^{-6}
		7	rs17168027	*	134099287	A	G	0.89	0.89	0.21	0.05	4.38x10^{-6}
		10	rs10825165		55354348	A	G	0.11	0.92	-0.22	0.05	3.11x10^{-6}
		15	rs9806467		48965568	C	T	0.83	1.11	-0.16	0.03	3.81x10^{-6}
	**Repeated Measures**	1	rs1188356		30983669	G	T	0.79	1.02	0.16	0.03	7.33x10^{-7}
		1	rs1413227		97477486	C	T	0.92	1.02	-0.22	0.05	3.19x10^{-6}
		2	rs13394665	*	64244935	A	C	0.73	0.97	0.15	0.03	6.70x10^{-7}
		2	rs1069256	*	173881973	C	T	0.75	1.02	0.17	0.03	6.00x10^{-7}
		3	rs9874245		81211967	A	G	0.85	1.03	0.20	0.04	1.63x10^{-7}
		4	rs1380155		128947268	A	G	0.95	1.91	0.45	0.10	4.22x10^{-6}
		7	rs17168027	*	134099287	A	G	0.89	0.89	0.22	0.05	1.98x10^{-6}
		10	rs11003972		55421987	C	G	0.85	0.98	0.19	0.04	2.09x10^{-6}
		15	rs1026672		64280411	C	T	0.81	0.98	0.16	0.03	4.10x10^{-6}
		15	rs8029458		89405378	G	T	0.80	0.93	-0.16	0.04	3.67x10^{-6}
		15	rs11630508		99138869	A	G	0.15	0.87	0.20	0.04	1.39x10^{-6}
**European**												
	**Baseline**	1	rs1543251		105839118	G	T	0.86	1.02	0.20	0.04	1.33x10^{-7}
		2	rs2602569		168998331	C	T	0.08	0.98	0.22	0.05	4.11x10^{-6}
		4	rs4399935		156175906	A	C	0.65	0.97	0.13	0.03	4.71x10^{-6}
		7	rs7457833	*	33521559	A	G	0.90	1.01	-0.20	0.04	3.26x10^{-6}
		8	rs1460396		2913482	A	G	0.19	0.89	0.16	0.03	4.90x10^{-6}
		10	rs6583751		92828756	C	G	0.61	0.96	0.13	0.03	2.48x10^{-6}
		13	rs1838113		18394504	C	T	0.07	0.81	-0.26	0.06	4.11x10^{-6}
		13	rs4884095		77898753	A	T	0.78	0.98	-0.15	0.03	9.09x10^{-7}
	**Averaged**	1	rs12564617		105832151	A	G	0.14	1.01	-0.15	0.03	1.71x10^{-6}

		3	rs17694541	*	135885739	C	T	0.08	1.06	-0.18	0.04	$4.91x10^{-6}$
		7	rs7457833	*	33521559	A	G	0.90	1.01	-0.18	0.04	$2.12x10^{-6}$
		9	rs10739282	*	111488202	A	C	0.41	0.99	0.11	0.02	$8.26x10^{-7}$
		10	rs12146350		22883117	C	G	0.40	0.97	-0.11	0.02	$3.48x10^{-6}$
	**Repeated Measures**	1	rs897632		2816023	A	G	0.23	1.00	-0.13	0.03	$2.51x10^{-6}$
		1	rs12563710		105831868	A	C	0.86	1.01	0.16	0.03	$1.30x10^{-6}$
		3	rs17694541	*	135885739	C	T	0.08	1.06	-0.19	0.04	$3.51x10^{-6}$
		7	rs4720629		5441935	C	T	0.91	1.01	0.19	0.04	$2.12x10^{-6}$
		7	rs7457833	*	33521559	A	G	0.90	1.01	-0.19	0.04	$7.93x10^{-7}$
		9	rs10739282	*	111488202	A	C	0.41	0.99	0.11	0.02	$8.40x10^{-7}$
		10	rs7916922		22859264	C	T	0.37	0.92	-0.11	0.02	$3.93x10^{-6}$
		10	rs11013046		22880589	C	T	0.41	1.00	-0.11	0.02	$6.04x10^{-7}$
		10	rs12267148		92850261	A	G	0.67	0.93	0.12	0.03	$1.52x10^{-6}$
**Chinese**												
	**Baseline**	10	rs12769343		29247812	A	G	0.33	0.92	-0.24	0.05	$2.48x10^{-6}$
	**Averaged**	11	rs2461672	*	80525681	C	T	0.79	0.90	0.25	0.05	$1.42x10^{-6}$
		10	rs4529793	*	65960699	A	G	0.09	1.02	0.33	0.07	$4.28x10^{-6}$
	**Repeated Measures**	2	rs12990176		150385322	G	T	0.06	0.96	-0.32	0.07	$3.53x10^{-6}$
		10	rs4529793	*	65960699	A	G	0.09	1.02	0.34	0.07	$2.71x10^{-7}$
		11	rs2461672	*	80525681	C	T	0.79	0.90	0.24	0.05	$3.89x10^{-6}$
		12	rs17115484		74370892	C	T	0.10	0.97	-0.29	0.06	$7.05x10^{-7}$
**Hispanic**												
	**Baseline**	2	rs17023193		83192390	A	T	0.12	1.03	-0.28	0.06	$3.94x10^{-7}$
		3	rs1127233	*	126109714	G	T	0.23	1.00	0.24	0.04	$3.85x10^{-8}$
		4	rs2903246		11750932	A	T	0.38	1.03	-0.18	0.04	$3.21x10^{-6}$
		4	rs4695747	*	175015856	A	G	0.93	0.99	0.39	0.07	$1.25x10^{-7}$
		5	rs806600		172847545	A	G	0.49	0.99	-0.17	0.04	$2.93x10^{-6}$
		7	rs909092		141759938	A	G	0.90	0.98	0.31	0.06	$7.03x10^{-7}$
	**Averaged**	4	rs4695747	*	175015856	A	G	0.93	0.99	0.28	0.06	$4.78x10^{-6}$
		5	rs7728557	*	74329715	A	G	0.32	1.03	0.15	0.03	$1.61x10^{-6}$
		14	rs8016794		52070396	A	G	0.83	1.06	0.18	0.04	$4.49x10^{-6}$
	**Repeated Measures**	2	SNP_A-1966287		191577187	C	T	0.11	1.03	0.24	0.05	$1.71x10^{-6}$
		3	rs1127233	*	126109714	G	T	0.23	1.00	0.18	0.04	$2.06x10^{-6}$
		4	rs4695747	*	175015856	A	G	0.93	0.99	0.31	0.06	$9.25x10^{-7}$
		5	rs7728557	*	74329715	A	G	0.32	1.03	0.16	0.03	$1.96x10^{-6}$

5	rs244897	167851370	C	T	0.11	0.92	0.24	0.05	$4.23x10^{-6}$
6	rs3805944	43153440	G	T	0.91	1.03	-0.24	0.05	$3.11x10^{-6}$
7	rs123778	157062905	C	G	0.85	1.31	-0.24	0.05	$2.43x10^{-6}$
14	rs7148635	66979360	A	G	0.59	1.02	-0.15	0.03	$4.25x10^{-6}$
14	rs17106996	77702071	A	G	0.92	0.95	-0.27	0.06	$1.03x10^{-6}$
16	rs9652625	51663724	C	G	0.68	0.89	-0.17	0.04	$2.73x10^{-6}$

*indicates SNP that reached genome-wide suggestive p-value in multiple approaches

MAF>5%, INFO > 0.80, LD R^2 > 0.80

CHR: chromosome, SNP: single nucleotide polymorphism, BP: base pair, A1/A2: coded and non-coded allele, FRQ: coded allele frequency, INFO: imputation information score, SE: standard error, MAF: minor allele frequency, LD: linkage disequilibrium
