# Supplementary material for: Comparative genome-wide association studies of a depressive symptom phenotype in a repeated measures setting by race/ethnicity in the multi-ethnic study of atherosclerosis
Source: BMC Genet. 2015 Oct 12;16:118. doi: 10.1186/s12863-015-0274-0 (PMC4603946; doi:10.1186/s12863-015-0274-0)
Supplement: Additional file 6: — Methodological information on anti-depressant adjustment. (PDF 269 kb) [file 12863_2015_274_MOESM6_ESM.pdf]

Adjustment for Anti-depressant use:

Rather than removing individuals taking anti-depressant medication and losing valuable genetic information, CES-D scores were adjusted for treatment effect using a similar algorithm used for adjustment of blood pressure for persons taking anti-hypertensive medications (Levy *et al.* 2000). Since response to anti-depressant medication is highly variable and information on compliance to medication is not always available in population-based studies, there were two assumptions made when adjusting CES-D scores. First, CES-D scores of anti-depressant users are right-censored. That is, the CES-D score while *on* anti-depressant medication is lower than the score while not taking anti-depressant medication. Second, participants with low depressive symptoms scores respond less to anti-depressant medication than persons with high depressive symptom scores, on average.

The algorithm for adjustment of anti-depressant use was run separately for multiple factors: gender, race, and exam period, on a total of 6,438 individuals. The nonparametric imputation algorithm replaces the CES-D score of a person using anti-depressants with the mean depressive symptom score for all persons taking anti-depressants with the same or higher depressive symptom score. This method has recently been used in a large depressive symptom GWAS consortium (Hek *et al.* 2013). Anti-depressant use was defined at each exam by self-reported monoamine oxidase inhibitor (i.e. isocarboxazid, phenylzine, tranylcypromine), tricyclic anti-depressant (i.e. amitriptyline, doxepin, nortriptyline) and/or non-tricyclic anti-depressant (i.e. citalopram, escitalopram, fluoxetine, sertraline) use coded as yes/no. Since those who had missing information on anti-depressant use were not significantly different on exam-specific mean CES-D scores than those who did not take anti-depressants (exam 1: p-value = 0.5955, exam 3: p-value = 0.1476, exam 4: p-value = 0.1103), individuals with missing information on anti-depressants were classified as “0 – not taking anti-depressants” for imputation purposes. This allowed the increase of sample sizes for each exam and thus an increase in statistical power. Missing information on anti-depressant use was observed on two participants from exam 1, 91 participants from exam 3, and 167 participants from exam 4. The distribution of CES-D in this sample is skewed right, with the majority of

values being less than 10. A total of 7.6%, 7.9% and 8.1% of persons were on anti-depressant medications at exams 1, 3 and 4, respectively.  We chose not to exclude participants taking anti-depressant medication as they often are individuals with depression or higher depressive symptom scores and thus add value to genetic studies.

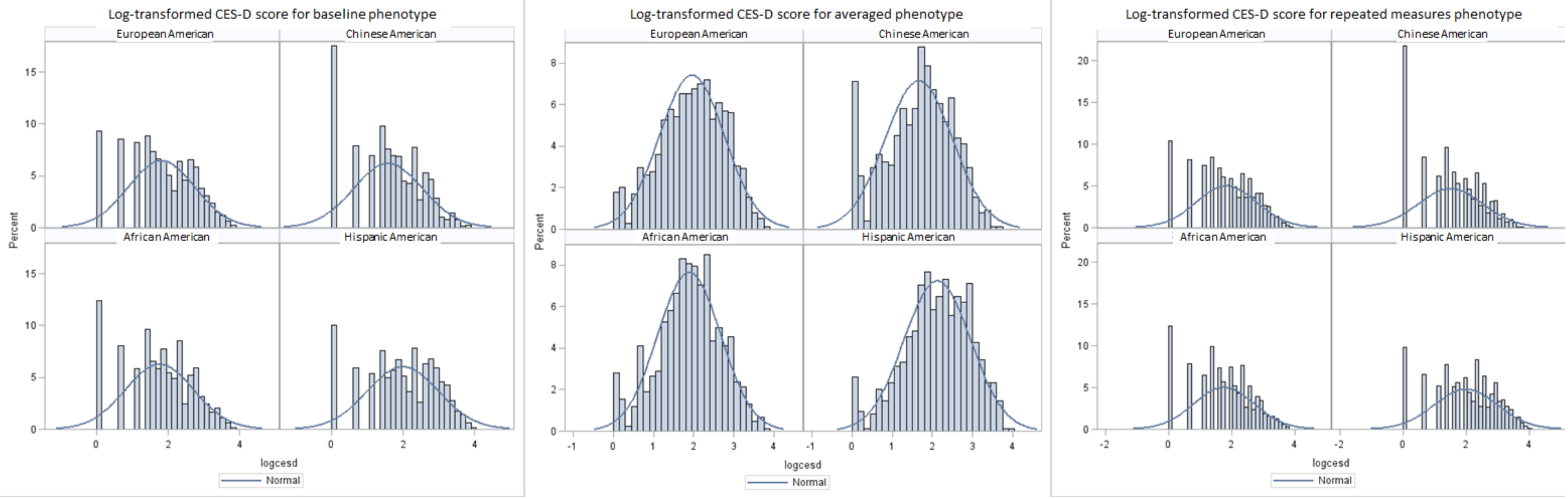
Log-transformed CES-D score for baseline phenotype
European American
Chinese American
African American
Hispanic American
Percent
logcesd
Normal
Log-transformed CES-D score for averaged phenotype
European American
Chinese American
African American
Hispanic American
Percent
logcesd
Normal
Log-transformed CES-D score for repeated measures phenotype
European American
Chinese American
African American
Hispanic American
Percent
logcesd
Normal

Resources

Hek K., Demirkan A., Lahti J., Terracciano A., Teumer A., Cornelis M.C., Amin N., Bakshis E., Baumert J., Ding J., Liu Y., Marciante K., Meirelles O., Nalls M.A., Sun Y.V., Vogelzangs N., Yu L., Bandinelli S., Benjamin E.J., Bennett D.A., Boomsma D., Cannas A., Coker L.H., de Geus E., De Jager P.L., Diez-Roux A.V., Purcell S., Hu F.B., Rimm E.B., Hunter D.J., Jensen M.K., Curhan G., Rice K., Penman A.D., Rotter J.I., Sotoodehnia N., Emeny R., Eriksson J.G., Evans D.A., Ferrucci L., Fornage M., Gudnason V., Hofman A., Illig T., Kardia S., Kelly-Hayes M., Koenen K., Kraft P., Kuningas M., Massaro J.M., Melzer D., Mulas A., Mulder C.L., Murray A., Oostra B.A., Palotie A., Penninx B., Petersmann A., Pilling L.C., Psaty B., Rawal R., Reiman E.M., Schulz A., Shulman J.M., Singleton A.B., Smith A.V., Sutin A.R., Uitterlinden A.G., Volzke H., Widen E., Yaffe K., Zonderman A.B., Cucca F., Harris T., Ladwig K.H., Llewellyn D.J., Raikkonen K., Tanaka T., van Duijn C.M., Grabe H.J., Launer L.J., Lunetta K.L., Mosley T.H., Jr., Newman A.B., Tiemeier H. & Murabito J. (2013) A Genome-Wide Association Study of Depressive Symptoms. *Biol Psychiatry*.

Levy D., DeStefano A.L., Larson M.G., O'Donnell C.J., Lifton R.P., Gavras H., Cupples L.A. & Myers R.H. (2000) Evidence for a gene influencing blood pressure on chromosome 17. Genome scan linkage results for longitudinal blood pressure phenotypes in subjects from the framingham heart study. *Hypertension* **36**, 477-83.
